# Patient and caregiver outcomes with levodopa-carbidopa intestinal gel in advanced Parkinson’s disease

**DOI:** 10.1038/s41531-021-00246-y

**Published:** 2021-11-30

**Authors:** Francesc Valldeoriola, María José Catalán, Francisco Escamilla-Sevilla, Eric Freire, Jesús Olivares, Esther Cubo, Diego Santos García, Matilde Calopa, Pablo Martínez-Martín, Juan Carlos Parra, Gloria Arroyo, José Matías Arbelo

**Affiliations:** 1 Movement Disorders Unit, Neurology Service, Clínic Hospital, 170, Villarroel St., 08036 Barcelona, Spain; 2 Neurology Service, Clínico San Carlos Hospital, Profesor Martín Lagos St., 28040 Madrid, Spain; 3 Movement Disorders Unit. Neurology Service, Virgen de las Nieves University Hospital, “Biohealth Investigation Institute. Ibs, Granada, Spain; 4 Neurology Service, Elche University General Hospital, 11, Camino Almazara St., 03203 Elche, Alicante Spain; 5 Neurology Service, Torrecárdenas Hospital Center, Hermandad de Donantes de Sangre St., 04009 Almería, Spain; 6 Neurology Service, Burgos University Hospital, 3, Islas Baleares Av., 09006 Burgos, Spain; 7 Department of Neurology, A Coruña University Hospital Center (CHUAC), A Coruña, Spain; 8 Neurology Service, Bellvitge University Hospital, Feixa Llarga St., 08907L’Hospitalet de Llobregat, Barcelona, Spain; 9 National Center of Epidemiology and Center for Networked Biomedical Research in Neurodegenerative Diseases (CIBERNED), Carlos III Institute of Health, 5, Monforte de Lemos Av., 28029 Madrid, Spain; 10 AbbVie Spain, S.L.U., 91, De Burgos Av., 28050 Madrid, Spain; 11 Neurology Service, Insular of Gran Canaria University Hospital, Marítima del Sur Av., 35016 Las Palmas, de Gran Canaria Spain

**Keywords:** Parkinson's disease, Outcomes research

## Abstract

Levodopa-carbidopa intestinal gel (LCIG) has shown to be efficacious in motor and non-motor symptoms (NMS). Nevertheless, studies with patient Quality of Life (QoL) as a primary endpoint are scarce. To assess the effect of LCIG on Advanced Parkinson’s Disease (APD) patients QoL. Secondarily, the impact on motor symptoms and NMS, emotional well-being, treatment satisfaction, and caregiver QoL, stress, disease burden, anxiety, depression, and work impairment were also investigated. In this prospective, 6-month multicenter postmarketing observational study, LCIG was administered to 59 patients with APD. Endpoints were assessed using validated scales and questionnaires. LCIG significantly improved patient QoL (PDQ-39 mean change ± standard deviation from baseline, −12.8 ± 14.6; *P* < 0.0001), motor symptoms (UPDRS-III in “On,” −6.5 ± 11.8; *P* = 0.0002), NMS (NMSS, −35.7 ± 31.1; *P* < 0.0001), mood (Norris/Bond-Lader VAS, −6.6 ± 21.1; *P* = 0.0297), fatigue (PFS-16, −0.6 ± 1.0; *P* = 0.0003), depression (BDI-II, −5.1 ± 9.4; *P* = 0.0002), anxiety (BAI, −6.2 ± 9.6; *P* < 0.0001), and patient treatment satisfaction (SATMED-Q, 16.1 ± 16.8; *P* < 0.0001). There were significant correlations between the change from baseline to 6 months between PDQ-39 and UPDRS-IV, NMSS, BAI, BDI-II, AS, and PFS-16 scores, and Norris/Bond-Lader alertness/sedation factor. Caregiver anxiety also improved (Goldberg anxiety scale, −1.1 ± 1.0; *P* = 0.0234), but the clinical relevance of this finding is questionable. The serious adverse events reported were similar to those previously described for LCIG. In patients with APD, LCIG improves QoL, motor symptoms and NMS, emotional well-being, and satisfaction with the treatment. Improvement in patient QoL is associated with improvements in motor complications, NMS, anxiety, depression, apathy and fatigue. Improvements in patients’ QoL does not correspond with improvements in caregivers’ QoL or burden.

## Introduction

Parkinson disease (PD) is characterized not only by the presence of “classical” motor symptomatology, but also by multiple non-motor symptoms (NMS) of different nature, which cause disability and impact quality of life (QoL), especially in the advanced stage of the disease^1^. At this stage, when oral medication no longer controls motor fluctuations but patients still respond to levodopa, three device-aided therapies (DATs) may be considered: deep brain stimulation (DBS), levodopa-carbidopa intestinal gel (LCIG) or apomorphine injection and infusion. LCIG and DBS have shown to improve patient’s QoL in clinical trials^2,3^, but no apomorphine that failed to show this effect in a placebo-controlled study^4^. LCIG is a valid option for most patients who are considered for DBS and for many of the patients for whom DBS is contraindicated. However, all 3 DATs have demonstrated significant improvements in motor fluctuations^2–4^. So, in the absence of randomized controlled clinical trials among them, the best available evidence (efficacy, contraindications and possible adverse reactions) should be combined with the professional’s expertise and the patient’s preferences to make a decision.

Health-related QoL is a patient-reported outcome considered to be a key measure of patient global status^1^, and should be the main purpose of any PD symptomatic treatment. Disease-specific instruments, such as the 39-item Parkinson’s Disease Questionnaire (PDQ-39)^5^, are valuable in assessing disease-specific problems and measuring the success of treatment in altering QoL over time^6^. The PDQ-39 has been shown to be feasible, reliable, valid, and responsive to change in patients with PD.

LCIG has demonstrated benefits controlling motor fluctuations and NMS in patients with advanced PD (APD) in randomized, controlled, clinical trials^2,7^ and observational studies^8–19^. QoL has been assessed in some of them^2,8,10–19^ as a secondary objective, showing significant improvements; however, data on the impact of LCIG on patient’s mood and behavior are scarce. In addition, in most of the studies the questionnaire used was the PDQ-8^8,10,12,14,15,17^. PDQ-8 is a short form of the PDQ-39 and, although it has been validated, it has the disadvantage that it does not offer the possibility to assess individual domains but just a simplified index and, for this reason, PDQ-8 is more convenient for using in clinical settings rather than in clinical research.

We conducted this study to assess, the effect of 6-month treatment with LCIG on the QoL of patients with APD using the self-reported PDQ-39. Additionally, we assessed patient’s NMS related to emotional well-being and satisfaction with treatment, as well as caregiver QoL, burden, and other symptoms/aspects related with his/her role as caregivers of a patient with APD. We hypothesized that LCIG will significantly improve the patient’s QoL, and this improvement will correlate with the improvements in motor and non-motor symptoms, and patient’s emotional well-being, as well as in the caregiver QoL.

## Results

### Baseline characteristics

Sixty-two patients and caregivers from 23 Spanish centers were enrolled; 59 were evaluable and constituted the intent-to-treat and safety populations. Three patients were non-evaluable because they did not reach the nasoduodenal test phase.

The mean age of patients was 67.9 ± 7.5 years and 61.0% were male; the mean age of caregivers was 58.8 ± 11.7 years and 64.4% were female. The clinical and sociodemographic characteristics of patients and caregivers are shown in Tables 1 and 2, respectively.

**Table 1:** Clinical and socio-demographic characteristics of the patients, at baseline.

Baseline characteristics	
Age, years (mean ± SD)	67.9 ± 7.5
Sex male, *n* (%)	36 (61.0)
Race Caucasian, *n* (%)	59 (100)
Marital status, *n* (%)	
Single	2 (3.4)
Married/Couple’s relationship	45 (76.3)
Separated/Divorced	5 (8.5)
Widower/Widow	7 (11.9)
Highest level of education, *n* (%)	
None	12 (22)
Primary school	34 (57.6)
Secondary school	3 (5.1)
Vocational education	3 (5.1)
University	6 (10.2)
Duration of the disease, years (mean ± SD)	12.7 ± 6.0
UPDRS-IV (mean ± SD)	3.6 ± 2.0
Hoehn & Yahr during “On”, *n* (%)	
Stage 1	27 (45.7)
Stage 2	25 (42.4)
Stage 3	6 (10.2)
Stage 4	1 (1.7)
Hoehn & Yahr during “Off”, *n* (%)	
Stage 1	1 (1.7)
Stage 2	8 (13.6)
Stage 3	35 (59.3)
Stage 4	15 (25.4)
Schawb&England ADL during “On” (mean ± SD)	70.3 ± 23.1
Schawb&England ADL during “Off” (mean ± SD)	31.0 ± 18.6
PDQ-39 (mean ± SD)	46.7 ± 13.6
UPDRS-III during “On” (mean ± SD)	30.1 ± 14.2
Off-time, h per day (mean ± SD)	5.8 ± 3.0
On-time with dyskinesias, h per day (mean ± SD)	4.6 ± 4.8
NMSS (mean ± SD)	83.2 ± 32.6
Norris/Bond-Lader VAS (mean ± SD)	42.6 ± 17.6
PFS-16 (mean ± SD)	3.7 ± 0.8
AS (mean ± SD)	11.4 ± 6.4
BDI-II (mean ± SD)	18.1 ± 9.7
BAI (mean ± SD)	19.8 ± 9.4
SATMED-Q (mean ± SD)	52.8 ± 15.7
Daily levodopa dose^a^, mg (mean ± SD)	1099.0 ± 538.2
Prior antiparkinsonian medication use, *n* (%)	
Dopamine agonist	59 (100)
COMT inhibitor	7 (11.9)
MAO-B inhibitor	24 (40.7)
Amantadine	12 (20.3)
Other	13 (22.0)

^a^Includes levodopa dose and levodopa equivalent daily dose of concomitant antiparkinsonian medications. *ADL* Activity of Daily Living, *AS* Apathy Scale, *BAI* Beck Anxiety Inventory, *BDI* Beck Depression Inventory, *COMT* Catechol-O-methyl transferase, *MAO-B* Monoamine oxidase B, *NMSS* Non-Motor Symptom Scale, *PDQ-39* Parkinson’s Disease Questionnaire 39-item, *PFS-16* Parkinson’s Fatigue Scale 16-item, *SATMED-Q* Satisfaction with the Medication Questionnaire, *UPDRS* Unified Parkinson’s Disease Rating Scale (part III, motor examination; part IV, motor complications), *VAS* Visual Analogue Scale.

**Table 2:** Baseline sociodemographic characteristics of caregivers and global scores of SQLC, ZBI, CSI, Goldberg Anxiety Scale, Goldberg Depression Scale, and WPAI.

Baseline characteristics	
Age, years*(mean ± SD)	58.8 ± 11.7
Sex (female), *n* (%)	38 (64.4)
Race (Caucasian),* n (%)	54 (91.5)
Marital status, *n* (%)	–
Single	4 (6.8)
Married/couple’s relationship	46 (78.0)
Separated/divorced	3 (5.1)
Widower/widow	1 (1.7)
Missing	5 (8.5)
Highest level of education, *n* (%)	
None	5 (8.5)
Primary school	23 (39.0)
Secondary school	9 (15.2)
Vocational education	5 (8.5)
University	12 (20.3)
Missing	5 (8.5)
Employment status, *n* (%)	
Never worked	6 (10.2)
Employed	13 (22.0)
Unemployed	6 (10.2)
Retired	16 (27.1)
On sick leave	3 (5.1)
Another situation	10 (16.9)
Missing	5 (8.5)
Full time care,*n (%)	25 (42.4)
SQLC* (mean ± SD)	63.6 ± 26.4
ZBI* (mean ± SD)	24.9 ± 13.5
CSI* (mean ± SD)	5.0 ± 3.3
Goldberg Anxiety Scale** (mean ± SD)	7.2 ± 1.3
Goldberg Depression Scale*** (mean ± SD)	5.7 ± 1.9
WPAI	
• Outcome score 1 (mean ± SD)	12.0 ± 27.5
• Outcome score 2 (mean ± SD)	26.4 ± 28.2
• Outcome score 3 (mean ± SD)	36.1 ± 33.9
• Outcome score 4 (mean ± SD)	25.6 ± 25.3

Missing subjects *n = 5; **n = 31; ***n = 34.

*APD* advanced Parkinson’s disease, *CSI* Caregiver Strain Index, *SQLC* Scales of Quality of Life for Caregivers, *WPAI* Work Productivity and Activity Impairment (assessed in 16 caregivers that were employed during the study; Outcome 1: percent work time missed due to APD; Outcome 2: percent impairment while working due to APD; Outcome 3: percent overall work impairment due to APD; and Outcome 4: percent activity impairment due to APD), *ZBI* Zarit Burden Inventory.

### Changes from baseline to last visit

The mean dose of LCIG administered to the patients increased from 75.52 ± 94.24 mL, at hospital discharge (after LCIG dose titration), to 84.45 ± 70.73 mL at the final visit, corresponding to 1510.33 ± 1,884.73 mg and 1689.00 ± 1,414.62 mg of levodopa, respectively. The mean LEDD increased from baseline (1099.04 ± 538.16 mg) to the final visit (1861.43 ± 1389.97 mg; *P* < 0.0002), the dopamine agonists LEDD gradually reduced from 225.18 ± 125.20 mg at baseline to 125.00 ± 77.01 mg at the final visit (*P* < 0.0001), and the rest of the antiparkinsonian drugs remain constant throughout the study.

**Fig. 1:**
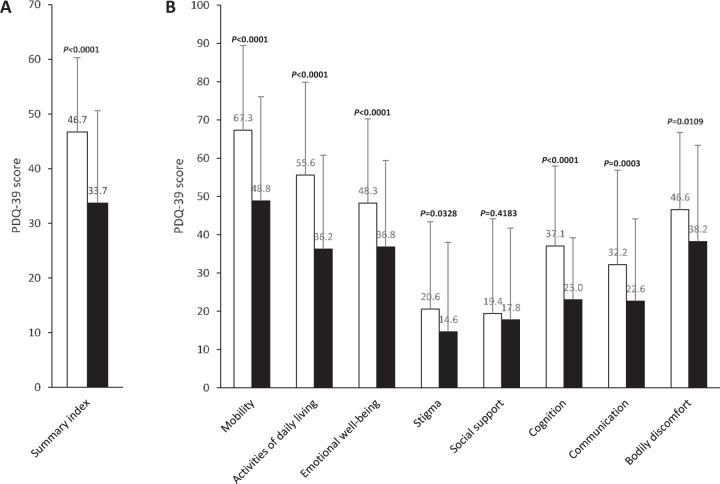
PDQ-39, 39-item Parkinson’s Disease Questionnaire. **A** Summary index. **B** Domain scores. White bars: data at baseline, black bars: data at final visit. *P* values for PDQ-39 score comparison between final visit and baseline. Values are given as mean ± SD.

**Fig. 2:**
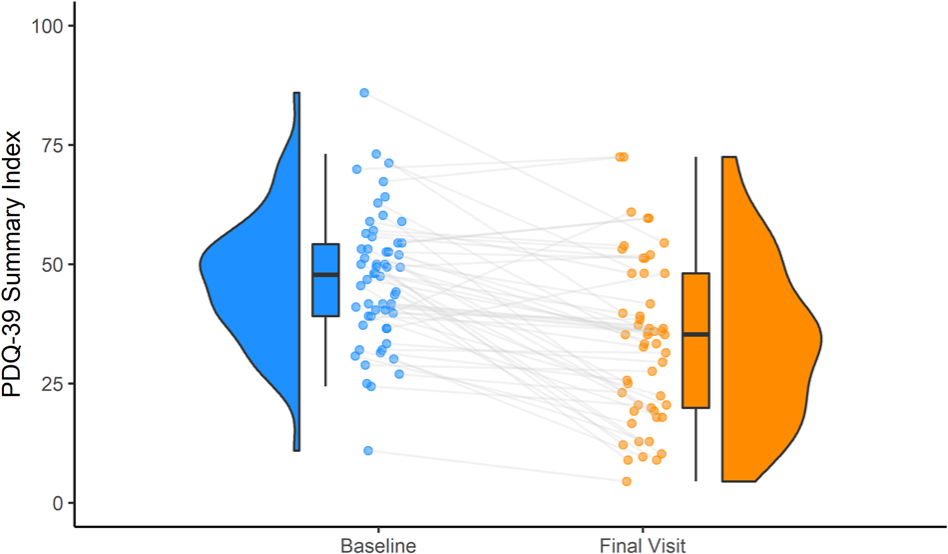
PDQ-39 summary index evolution from baseline to final visit. Violin plots representing the frequency and box plots representing the interquartile range containing 50% of the data, the median and the 95% confidence interval.

Patients´ QoL, represented by the PDQ-39 Summary Index as a whole, significantly improved throughout the study (Figs. 1 and 2). Changes were also statistically significant in all PDQ-39 domains, except in the “social support” domain.

**Fig. 3:**
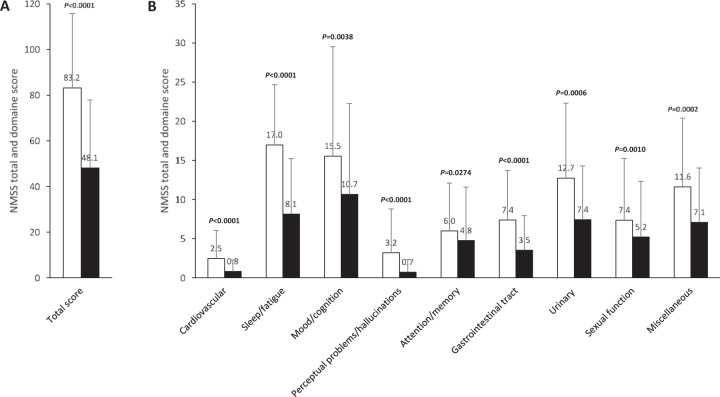
NMSS, Nonmotor symptom scale. **A** Total score. **B** Domain scores. White bars: data at baseline, black bars: data at final visit. P-values for NMSS score comparison between final visit and baseline. Values are given as mean ± SD.

**Fig. 4:**
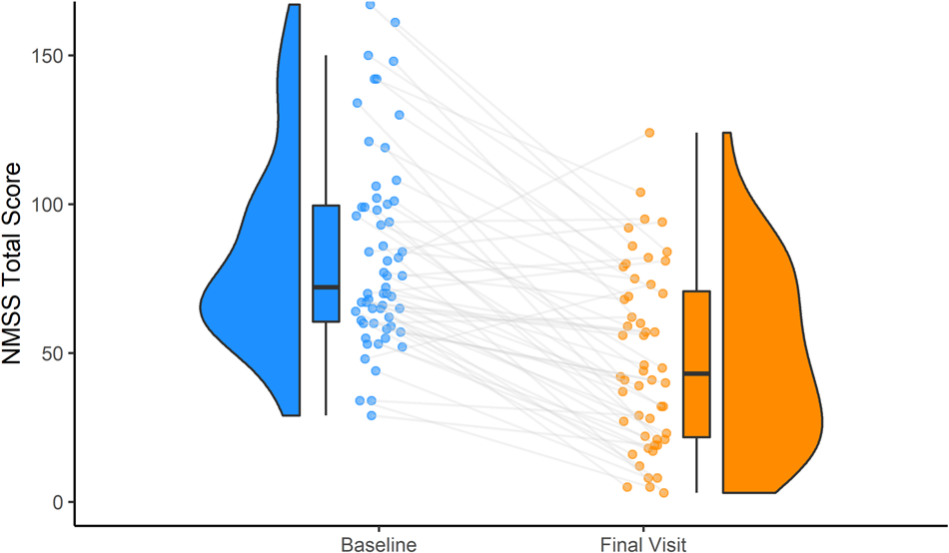
NMSS total score evolution from baseline to final visit. Violin plots representing the frequency and box plots representing the interquartile range containing 50% of the data, the median and the 95% confidence interval.

Compared with baseline scores, motor symptoms (UPDRS III) and motor complications (UPDRS IV) were also significantly improved throughout the study visits. The mean daily hours of “Off” time were reduced from 5.78 ± 3.00 to 2.34 ± 2.84 at the final visit (*P* < 0.0001). The mean total daily “On” time with dyskinesia decreased from 4.6 ± 4.8 at baseline to 3.5 ± 4.0 at the final visit although this reduction didn’t reach the statistical significance level (*P* = 0.077). The mean UPDRS-IV score was reduced from 3.58 ± 1.97 to 1.47 ± 1.31 at the final visit (*P* < 0.0001). There were no statistical differences in activities of daily living (ADL) assessed using the S&E scale in the “On” state (from 70.3 ± 23.1 to 75.5 ± 18.2; *P* = 0.29). NMS improved after 6 months of treatment with LCIG. Changes in NMSS scores, both total score and all domain scores, were significantly improved from baseline to the final visit (difference, 35.75 ± 31.12, *P* < 0.0001; percentage relative change, 41.41 ± 34.22, *P* < 0.0001; Figs. 3 and 4). Sleep/fatigue and gastrointestinal domains were the most improved domains (percentage relative change, 51.51 ± 39.52 and 34.01 ± 62.90, respectively).

At the end of the study, patients had a statistically significant positive change in mood. When the Norris/Bond-Lader factors were analyzed, there was a statistically significant improvement in alertness/sedation and calmness/relaxation, with mean scores changing from 44.8 ± 19.7 to 37.7 ± 17.3 (*P* = 0.028) and from 53.9 ± 23.2 to 45.2 ± 23.6 (*P* = 0.005), respectively. The improvement observed in the content/discontent factor (from 34.2 ± 18.5 to 30.9 ± 17.8) was not significant (*P* = 0.27). By contrast, no significant differences were observed in caregivers’ outcomes, except for anxiety.

Table 3 shows the mean changes observed in all primary and secondary endpoints. The comparisons of primary and secondary variables per visits (mixed model) were not statistically significant.

**Table 3:** Mean change ± SD from baseline to final visit (6 months ± 15 days) in the primary and secondary study endpoints (final score—baseline score).

Primary variable	*n*	Baseline	*n*	Final	*n*	Relative change (%)	*P* value	Effect size (CI 95%)
PDQ-39	58	46.7 ± 13.6	53	33.7 ± 16.9	52	−27.3 ± 30.6	<0.0001	0.87 (0.55,1.19)
**Secondary variables (patients)**								
UPDRS-III (ON)	59	30.1 ± 14.2	53	22.9 ± 11.6	53	−22.7 ± 23.6	0.0002	0.55 (0.26,0.84)
NMSS total	59	83.2 ± 32.6	52	48.1 ± 29.8	52	−41.4 ± 34.2	<0.0001	1.15 (0.79,1.50)
VAS Norris/Bond-Lader total	57	42.6 ± 17.6	53	36.6 ± 16.6	51	−12.9 ± 17.1	0.0297	0.31 (0.03,0.59)
PFS-16	57	3.77 ± 0.77	53	3.11 ± 0.90	51	−12.7 ± 17.7	0.0003	0.55 (0.25,0.84)
AS	58	11.4 ± 6.40	53	12.3 ± 6.52	52	0.5 ± 6.9	0.5877	−0.08 (−0.20,0.35)
BDI-II	58	18.1 ± 9.75	53	13.2 ± 10.2	52	−31.3 ± 32.7	0.0002	0.55 (0.25,0.84)
BAI	58	19.8 ± 9.36	53	13.8 ± 10.1	52	−26.9 ± 27.1	<0.0001	0.65 (0.35,0.95)
SATMED-Q	58	52.8 ± 15.7	53	68.9 ± 11.9	52	26.3 ± 16.8	<0.0001	−0.96 (−0.63, −1.29)
**Secondary variables (caregivers)**								
SQLC total	54	63.6 ± 26.4	48	66.1 ± 28.7	47	1.7 ± 1.6	0.3126	−0.15 (−0.44,0.14)
ZBI	54	24.9 ± 13.6	48	24.4 ± 14.3	47	−0.4 ± 8.7	0.8321	0.03 (−0.26,0.32)
CSI	54	5.02 ± 3.33	48	4.46 ± 3.25	47	−4.2 ± 9.0	0.1945	0.20 (−0.14,0.38)
Goldberg depression scale	25	5.68 ± 1.95	13	6.00 ± 1.41	10	2.2 ± 2.3	0.7937	–
Goldberg anxiety scale	28	7.18 ± 1.33	15	6.40 ± 1.24	10	−12.9 ± 12.8	0.0234	–
WPAI								
Outcome score 1 (mean ± SD)	16	12.0 ± 27.5	10	10.6 ± 14.0	9	−1.4 ± 0.7	0.5222	–
Outcome score 2 (mean ± SD)	16	26.4 ± 28.2	10	15.4 ± 18.1	9	−10.8 ± 12.4	1.0000	–
Outcome score 3 (mean ± SD)	16	36.1 ± 33.9	10	25.4 ± 21.8	9	−10.7 ± 12.3	0.6481	–
Outcome score 4 (mean ± SD)	16	25.6 ± 25.3	10	24.0 ± 17.1	9	−1.6 ± 2.9	0.6741	–

*UPDRS* Unified Parkinson’s Disease Rating Scale (part III, motor examination), *ADL* Activity of Daily Living, *PDQ-39* Parkinson’s Disease Questionnaire 39-item, *NMSS* Nonmotor Symptom Scale, *VAS* Visual Analogue Scale, *PFS-16* Parkinson’s Fatigue Scale 16-item, *AS* Apathy Scale, *BDI* Beck Depression Inventory, *BAI* Beck Anxiety Inventory, *SATMED-Q* Satisfaction with the Medication Questionnaire, *SQLC* Scales of Quality of Life for Caregivers, *ZBI* Zarit Burden Inventory, *CSI* Caregiver Strain Index, *WPAI* Work Productivity And Activity Impairment (Outcome 1: percent work time missed due to APD; Outcome 2: percent impairment while working due to APD; Outcome 3: percent overall work impairment due to APD; and Outcome 4: percent activity impairment due to APD).

Due to the low number of valid data, the effect size of some of the scales/questionnaires haven’t been calculated.

At baseline, 27.1% of patients (16/59) were also receiving concomitant treatments for PD-related symptoms, including anxiety (*n* = 14), depression (*n* = 12), insomnia (*n* = 6), psychosis (*n* = 5), constipation (*n* = 5), or pain (*n* = 4). At the end of the study, half of these patients discontinued ≥1 concomitant treatment. In general, there was a reduction in all concomitant medications decreasing in a 19.0, 18.5, 16.7, 14.3, and 12.5 in anxiolytic, antidepressant, antipsychotic, insomnia, and constipation drug uses, respectively. No pain treatments were discontinued during the study.

### Correlations related with Quality of Life improvements

Significant correlations were observed between improvement in patient QoL and improvements in motor complications, NMS, anxiety, depression, apathy, fatigue, and the Norris/Bond-Lader alertness/sedation factor. After consideration of multicollinearity of possible independent predictors, a multivariate regression analysis showed that the variables that had the highest contributions to the model were PFS-16, NMSS, and SQLC (adjusted *R*^2^ = 0.43, *F* = 9.41, *P* < 0.001) (Table 4).

**Table 4:** Multivariate analysis with PDQ-39 as dependent variable and the most relevant predictor variables as independent variables.

Variable	with Variable	Coefficient	Standard Error	*P* value
PDQ-39	NMSS total	0.17	0.06	0.0072
PDQ-39	PFS-16	4.91	1.96	0.0166
PDQ-39	BDI-II	0.21	0.24	0.3911
PDQ-39	SQCL	−0.21	0.24	0.0148

*PDQ-39* Parkinson’s Disease Questionnaire 39-item, *NMSS* Nonmotor symptom scale, *PFS-16* Parkinson’s fatigue scale 16-item, *BDI-II* Beck depression inventory-II, *SQCL* Scale of Quality of Life of Caregivers.

### Safety data

Overall, 13 patients reported 18 SAEs. Three were reported in the nasoduodenal phase of study; of these SAEs, only 1 (pneumoperitoneum) was related to LCIG use. In the post-PEG phase, 15 SAEs were reported, seven were related to LCIG (ventricular tachycardia, gastrointestinal ulcer, paralytic ileus, pneumoperitoneum, infection, peripheral sensory neuropathy, and substance-induced psychotic disorder). Six (10.17%) out of the 59 patients prematurely discontinued the study, three during the nasoduodenal test phase (due to lack of efficacy in two cases, and due to a very narrow therapeutic margin in one case); and three after the PEG intervention phase (in one case due to lack of efficacy, due to exitus in other case, and due to patient’s decision in the third case). In addition, none of deaths were related to LCIG.

## Discussion

In our study, after 6 months of treatment with LCIG administered in daily clinical practice, the QoL of patients with APD improved significantly, which is consistent with results obtained in other interventional^2,7^ and observational^8–17,20–34^ studies in which QoL was assessed as a secondary endpoint. There was a 28% reduction in PDQ-39 scores after 6 months of treatment, which is a relevant result; the conclusions of the Society for Medical Decision-Making states that patients appear to be able to detect and value benefits when changes are >7 to 10% on QoL instruments or pain scales^35^. In our study, there was a statistically significant improvement in all domains except social support, which is in line with results from previous studies. Interestingly, social support only improved significantly in the Zibetti et al. study; 7 of 17 participants in this study had probable dementia, and the improvement in the social support domain was prominent in this subgroup of patients^33^. The results of the studies of patients receiving LCIG treatment are similar to the results for other second-line therapies (SLTs) in PD. Results from the OPTIPUMP study, which was similar to the ADEQUA study in terms of population and design, for patients receiving continuous subcutaneous apomorphine infusion, noted a reduction in the total score of the PDQ-39, although half of the domains did not have statistically significant reductions (4 out of 8), with social support being one of them^36^. Recently, Dafsari et al. investigated whether results in QoL outcomes after bilateral subthalamic nucleus deep brain stimulation are dependent on age. In this study, an improvement in the social support domain of the PDQ-39 was only observed in the youngest subgroup of patients (≤59 years old)^37^.

When motor symptoms and motor complications were assessed in our study, a significant improvement was noted in UPDRS III and IV scores, as observed in other studies^7,8,11–13,15–17,20,21,24,26,29,30,32,33,38^. Similarly, regarding NMS, there was a statistically significant improvement in NMSS total score in our study that corresponds with the results of other studies, in which NMSS total score improved significantly^8,10–16,34,38^. We also saw a significant improvement in all NMSS domains. When compared with other studies, sleep/fatigue and gastrointestinal tract were the NMSS domains that most frequently showed a statistically significant improvement (seven out of nine)^8,11,12,14–16,34^. In our study, the greatest benefit from LCIG treatment was observed in the NMSS score, which could explain the relevant result we found in PDQ-39 score^6^.

Treatment with LCIG also had a positive impact in patient emotional well-being, with improvements in mood, fatigue, depression, and anxiety. There were no statistically significant effects in apathy after 6 months of treatment. This could be related to dopamine agonist withdrawal^39^. It has been showed that depression^40–44^, fatigue^41,45–48^, apathy^45,49^, anxiety^42,50^, and mood^46,51,52^ have an impact on QoL in patients with PD; thus, it is not surprising that the improvements in these symptoms observed in our study correspond with the improvement noted in patient QoL.

Thus, patients in this study generally improved in relation to all aspects of APD. However, there were no statistically significant improvements in ADL as measured using the S&E scale, although improvements of the ADL domain in the PDQ-39 were noted. The S&E scale has been used extensively in patients with PD in recent years. While the clinimetric properties of this scale have never been established^53^, the International Parkinson and Movement Disorder Society Task Force in 2016 recommended its use in PD, both for clinical and research purposes^54^. Although the inter-rater reliability between patient and physician ratings seems to be high^54^, in our study, the S&E scale was completed by the neurologist. It is possible, therefore, that the physician rating could differ from the patient rating, based on results observed in the ADL domain of the PDQ-39. It should be noted that the PDQ-39 requested information from the past month (“Off” and “On”), whereas the S&E scale was evaluated at baseline and the final visit in the “On” state.

Special attention should be given to the caregiver results. We did not find a statistically significant effect in caregiver QoL, which is in concordance with the results of Sensi et al.^17^, but in contradiction with the results of Ciurleo et al.^19^. Surprisingly, except for anxiety, there was no improvement in a single scale in caregiver status. The stress index and depression of caregivers were unaffected. This is somewhat striking, as patient’s depression has been found to be related to caregiver’s depression^55^. Regarding caregiver’s anxiety, despite the fact that the reduction observed in the Goldberg anxiety scale was statistically significant, clinical relevance might be minimal as the final total score (6.4) was still above the cut-off point for anxiety (≥4). In some studies, caregiver’s burden improved;^19,24,31^ in others, no improvement was noted^2,11,18^. In our study, the ZBI score was low at baseline; therefore, there was little margin for improvement. This may be related to the exclusion of patients with dementia, a factor that contributes negatively to caregiver burden^56,57^. Similarly, other studies have not observed improvements in caregiver’s burden in patients with APD treated with an SLT^58^. There was also no improvement in WPAI scores in caregivers due to patient disability. There could be a few hypotheses of why patients improved motor symptoms, NMS, emotional well-being, and QoL but caregivers seemed not to be benefit from it. First, it is possible that the sample size for caregivers was too small to provide the appropriate statistical power (specifically for the WPAI questionnaire, where the number of currently employed caregivers was only 16 [of 59 caregivers in the study]). Another possible explanation was that study period (6 months) was not long enough of a timeline for caregivers of chronic, disabled, and advanced patients to observe a change (a statistically significant effect has been found in prospective long-term studies^24,31^), when a strong relation of dependence, with certain resistance to change, has already been established between the patient and the caregiver. If this is the case, it should not be assumed that an improvement in the quality of life of patients with advanced Parkinson’s disease corresponds to an immediate improvement in the quality of life of their caregivers. Finally, LCIG is an invasive treatment and a device-aid therapy that requires care and learning, which could negatively impact the caregiver, mainly during the first months that follow the beginning of LCIG therapy. The fact that patients receiving LCIG infusions are more active from the motor point of view probably requires more intense caregiver attention. All these factors could cause difficulties for caregivers after the PEG procedure at home. We should not forget that, based on Santos-García et al. study^59^, the main factors contributing to burden and stress in caregivers are mood (BDI-II) and ADL, which improved in our study according to the PDQ-39, but remained unchanged in the S&E scale.

Improvements in patient QoL (PDQ-39) correlated with improvements in UPDRS-IV; NMSS total score; BAI, BDI-II, AS, and PFS scores; and the Norris/Bond-Lader alertness/sedation factor, though the correlations were not strong. Nevertheless, none of the improvements observed in patient variables correlated with any of the caregiver variables, which is in contrast with the results obtained by Santos-García et al., which found a correlation between PDQ-39 scores and caregiver burden (ZBI)^31^. In the post hoc multivariate analysis, the QoL questionnaire for caregivers (SQLC) was one of the main variables that showed greater relation to the improvement in patient QoL (PDQ-39)^31^.

This study provides important clinical data related to the use of LCIG and how treatment improves motor symptoms, NMS, and overall QoL in patients with APD. This study also showed improvement in fatigue, depression, and anxiety in non-selected patients with PD under routine care treated with LCIG, using validated questionnaires. One of the study’s strengths was the assessments on caregivers. There are also inherent limitations associated with this study’s design, namely that it was an observational non-controlled study. Firstly, the potential contribution of unknown/unmeasured confounding factors when the correlation between patient and caregiver QoL was assessed against the other variables. Secondly the potential contribution of a placebo effect overestimating response, particularly when subjective variables are analyzed. Thirdly, the QoL evaluation was assessed using a health-related QoL tool, which was not accompanied by a generic QoL tool, such as the EuroQoL-5 Dimensions questionnaire. And, finally, although per inclusion criteria all patients at baseline had to score at least 26 at the MMSE, an even mild cognitive decline might have affected the reported outcomes.

To end, the safety profile of LCIG described in this study and the rate of discontinuations are comparable to the data published previously in the literature^2,8^ and collected in the Summary of Product Characteristics. In our study, 13 out of 62 patients reported 18 SAEs, of those, only 8 SAEs were related with LCIG and of those 5 SAEs were related to the percutaneous endoscopic gastrostomy. To this regard, comparisons with other SLTs are difficult to make due to different studies methodologies and follow-up periods. However, we can find that most of the SAEs reported by patients treated with SLTs are related to the surgery or the device. Thus, in the study of Dafsari et al. in which 54 patients who underwent bilateral STN-DBS and were followed-up for an approximately 5 months after surgery, 5 patients reported SAEs related to surgery or device or to stimulation^37^. Regarding apomorphine pump, in the study of Drapier et al. 143 patients were enrolled, of them 42 patients withdrew from the study due to drug intolerance, lack of efficacy or other reasons. Out of 100 patients who finally completed the study and had available data at 6 months, 13 reported 17 SAEs, being the most common those affecting patients’ skin^36^.

In summary, 6-month treatment with LCIG administered in routine clinical practice improved the QoL of patients with APD, as well as motor symptoms and NMS, emotional well-being, and caregiver anxiety. Improvements in PDQ-39 were associated with improvements in UPDRS-IV, NMSS, BAI, BDI-I, AS, and PFS-16 scores, and the Norris/Bond-Lader alertness/sedation factor. However, patients’ QoL improvements do not correspond with improvements in caregivers’ burden or caregivers’ QoL. Further studies focused on the correlation between patients’ motor symptoms, non-motor symptoms, and QoL with burden and QoL of caregivers may be warranted.

## Methods

ADEQUA was a multicenter, postmarketing, observational, prospective, 6-month single-arm study conducted with APD patients treated with LCIG, prescribed in routine clinical practice in accordance with the terms of the local marketing authorization, from October 2014 to November 2016 at 23 Spanish hospitals.

The study was conducted in accordance with the ethical principles that have their origin in the Declaration of Helsinki, with the protocol, and with standard operating procedures that guaranteed compliance with Good Clinical Practice, as described in the ICH guidelines. Following local regulations, the study was evaluated by the Spanish Agency of Medicines and Medical Devices and was approved by the Spanish Autonomous Communities and the ethics committees of the participating hospitals. All patients provided written informed consent before enrollment in the study.

### Patients

Eligible participants were outpatients aged ≥18 years with advanced levodopa-responsive PD with a diagnosis of idiopathic PD according to the UK Parkinson’s Disease Society Brain Bank Diagnosis Criteria^60^, with at least 2 h of daily “Off” time or 2 h of daily dyskinesia (assessed following MDS UPDRS-IV instructions). All patients had no dementia criteria, and had a Mini-Mental State Examination score ≥26, following Movement Disorders Society taskforce on dementia in PD recommendations^61^. Patients were excluded if they had any LCIG’s contraindication included in the Summary of Product Characteristics or product label. Information about the principal caregiver was collected when possible. The principal caregiver was the person in charge of the patient’s care most of the daytime.

Efficacy assessments were collected at baseline before LCIG treatment initiation with temporary nasojejunal (concomitant PD medications were administered at the discretion of treating physician), at discharge from hospital following Percutaneous Endoscopy Gastrostomy (PEG) placement, and at months 1, 3, and 6.

### Assessments

The primary endpoint was the mean change in PDQ-39^62^ score from baseline (before LCIG initiation at PEG placement) to final visit (month 6 ± 15 days after hospital discharge). PDQ-39 summary index was standardized from 0 to 100, with a lower summary index indicating a better QoL.

Secondary endpoints for patients included the mean change from baseline to final visit in i) Unified Parkinson’s Disease Rating Scale-Part III (UPDRS-III) score to evaluate motor impairment, measured during the best “On” time;^63^ ii) Non-Motor Symptoms Scale (NMSS) score;^64^ iii) Norris/Bond-Lader Visual Analog Scale (VAS) score (to evaluate the patient’s emotional well-being^65^); iv) 16-item Parkinson’s Fatigue Scale (PFS-16) score (cut-off of ≥3.30 was used to identify those perceiving fatigue to be a problem^66^); v) Apathy Scale (AS) score (patients with AS scores ≥14 were considered apathetic^67^); vi) Beck Depression Inventory-II (BDI-II) score;^68^ vii) Beck Anxiety Inventory (BAI) score;^69^ and viii) Treatment Satisfaction with Medicines-Questionnaire (SATMED-Q) score^70^.

For caregivers, endpoints included the mean change from baseline to final visit in i) Scale of Quality of Life of Caregivers (SQLC) score;^71^ ii) Zarit Burden Inventory (ZBI) score;^72^ iii) Caregiver Strain Index score;^73^ iv) Goldberg Anxiety and Depression Scale score;^74,75^ and v) Work Productivity and Activity Impairment (WPAI) score^76^.

### Safety measures

Safety data were collected using ad-hoc forms for Serious Adverse Events (SAEs). The physician notified AbbVie (as the sponsor of the study) within 24 h of the physician becoming aware of the event. An adverse event was considered serious if resulted in death, was life threatening, required inpatient hospitalization or prolongation of existing hospitalization, resulted in persistent or significant disability/incapacity or caused congenital anomaly/birth defect. Nonserious adverse events were not collected for analysis and were communicated by the clinicians following usual clinical practice.

### Statistical analysis

For sample size calculation, a minimal difference was assumed to detect 10% over a baseline score of 75.0 points in PDQ-39 based on the study of Lezcano et al.^77^. Assuming a standard deviation of 18.3^77^, accepting an alpha risk of 0.05 and beta risk of 0.2 in a two-sided, 48 patients were needed to detect a difference equal to or greater than 7.5 units. Assuming a loss to follow up rate of 23% 59.2 patients are needed, so 60 patients are needed. Sample size calculation was performed with v GRANMO. 7.10. Statistical analyses were conducted using SAS® V9.4 software (SAS Institute, Cary, NC). For all analyses, a significance alpha (α) value equal to 0.05 was assumed. Continuous variables are presented as means and standard deviations (SD). The Shapiro-Wilk test was used to test whether variables followed a normal distribution. Categorical variables are reported using frequencies and percentages. Confidence intervals (95%) for the percentage were calculated, when required. The magnitude of change is represented as the relative change and paired Cohen’s effect size (0.20–0.49 = small effect; 0.50–0.79 = moderate effect; and 0.80 = large effect).

Descriptive statistics were calculated for demographic and clinical characteristics. Contrast statistics were used for intra-patient comparisons using repeated measures. In the case of continuous variables, the repeated measures Student *t* test or the Wilcoxon rank-sum test was used, depending on the normality of the data. In the case of binary qualitative variables, the chi-square test or the McNemar test was used. A mixed model of repeated measures was used for comparisons between visits.

Univariate analysis and multivariate linear regression analysis were used to assess the association between the change in PDQ-39 (dependent variable) and other variables (independent variables) UPDRS III, Unified Parkinson’s Disease Rating Scale-Part IV (UPDRS IV), NMSS, S&E, VAS, PFS-16, AS, BDI-II, BAI, SATMED-Q, SQCL and ZBI from baseline to 6 months (±15 days) after hospital discharge. Correlations have been evaluated using Pearson test (in case of continuous variables) or Spearman test (in case of ordinal variables). Those independent variables with *P* < 0.1 in the univariate analysis (all of them except UPDRS III, S&E and ZBI) without multicollinearity were included in the multivariate linear regression model. Statistical analyses were performed with the intent-to-treat and safety populations. Levodopa equivalent daily dose (LEDD), including levodopa daily dose plus all antiparkinsonian therapies, were calculated according to Tomlinson et al.^78^.

### Study populations

The statistical analyses were performed in the following study populations:

- Per Protocol population (PP): included those patients (and caregivers) who met all inclusion criteria and were classified as suitable on the levodopa/carbidopa continuous nasoduodenal catheter infusion test. Those patients had to complete the 6-month period of treatment, and at least, they had the quality of life data collected with the PDQ-39 questionnaire at baseline and after 6 months (±15 days) of treatment.
- Intent-To-Treat (ITT) and Safety population: included those patients (and caregivers) that made up the PP population, those who were classified as not suitable on the levodopa/carbidopa continuous nasoduodenal catheter infusion test, and those that after undergoing the percutaneous endoscopic gastrostomy did not complete the 6-month treatment period.

As no significant differences were found between the two populations, only the results obtained in the ITT population are presented.

### Reporting Summary

Further information on research design is available in the Nature Research Reporting Summary linked to this article.

## Supplementary information

Reporting Summary

## Data Availability

AbbVie is committed to responsible data sharing regarding the clinical trials we sponsor. This includes access to anonymized, individual and trial-level data (analysis data sets), as well as other information (e.g., protocols and Clinical Study Reports), as long as the trials are not part of an ongoing or planned regulatory submission. This includes requests for clinical trial data for unlicensed products and indications. This clinical trial data can be requested by any qualified researchers who engage in rigorous, independent scientific research, and will be provided following review and approval of a research proposal and Statistical Analysis Plan (SAP) and execution of a Data Sharing Agreement (DSA). Data requests can be submitted at any time and the data will be accessible for 12 months, with possible extensions considered. For more information on the process, or to submit a request, visit the following link: https://www.abbvie.com/our-science/clinical-trials/clinical-trials-data-and-information-sharing/data-and-information-sharing-with-qualified-researchers.html.
